# An Investigation of Hyperostosis Frontalis Interna in a Modern Anatomical Body Donor Population

**DOI:** 10.1002/ca.70025

**Published:** 2025-09-10

**Authors:** Amy C. Beresheim, Amanda Hall

**Affiliations:** ^1^ Department of Anatomy and Cell Biology Rush University Medical Center Chicago Illinois USA; ^2^ Department of Communication Disorders and Sciences Rush University Medical Center Chicago Illinois USA

**Keywords:** anatomical body donation, body donor demographics cause of death analysis, hyperostosis frontalis interna, neoplasms, structural vulnerability index

## Abstract

This research sought to examine the prevalence and severity of hyperostosis frontalis interna (HFI) in the Chicagoland anatomical body donor population. The study further aimed to elucidate potential demographic risk factors for HFI, including sex, age at death, and structural vulnerability index (SVI), as well as any common comorbidities, as gleaned from death certificates. HFI is an irregular bony overgrowth of the endocranial surface of the frontal bone. It is most often observed in postmenopausal women or in individuals with growth hormone disorders. This work investigated the distribution of HFI in a predominantly geriatric anatomical body donor population (*n*_total_ = 235, *n*_female_ = 127 *n*_male_ = 108; 19–104 years), using a macroscopic classification system that considers both the morphological appearance and the size of the affected area. Relationships between HFI and variables of interest were assessed through various non‐parametric statistical tests and binomial logistic regression. While HFI was not associated with age‐at‐death or SVI, results indicate that there were significant sex differences in both HFI prevalence and severity. Females demonstrated higher rates of HFI across all severity types, whereas in males, HFI lesions were much less common and mostly limited to the earliest stages of disease progression. HFI was also associated with neoplasms as a cause of death. Among cancer deaths, individuals with hormone‐sensitive cancers had a higher prevalence of HFI, but this difference was not statistically significant. While the causal pathways of these relationships remain unclear, the association with cancer may potentially explain the reportedly higher HFI prevalence rates in modern compared to past populations. Moreover, this research has bioarcheological and forensic implications as HFI is sometimes used to infer age and sex, given its association with older‐aged females.

## 1 Introduction

Hyperostosis frontalis interna (HFI) involves the bilateral thickening of the endocranial surface of the frontal bones through the formation of multiple bony nodules (Hershkovitz et al. 1999). It does not appear to affect either the diploë or the outer bone table, although this is somewhat contentious (Lynnerup et al. 2005; She and Szakacs 2004; Hasegawa et al. 1983; Moore 1935; Rühli et al. 2004; Prescher and Adler 1993), and HFI lesions seldom cross suture lines. HFI is typically asymptomatic, and often found incidentally during medical imaging (Hasegawa et al. 1983; Kocabas et al. 2008; Ramchandren and Liebeskind 2007; Khizar et al. 2023; Chaljub et al. 1999; Win and Aparici 2012; Byrne et al. 2020; Waclawik 2006), autopsy (She and Szakacs 2004; Bascou et al. 2019; Cvetković et al. 2019; Devriendt et al. 2005; Nikolić et al. 2010), or cadaveric dissection (Alvarez et al. 2022; Stiene and Frank 2022; Talarico et al. 2008). The etiology and pathogenesis of HFI remain elusive, but HFI is largely believed to be associated with endocrine disruption (Kollin and Fehér 1986; Rudali 1968). Prevalence rates vary markedly by study, ranging anywhere from 0% to 87% (Table 1). Although more commonly found in older individuals, HFI has been observed in those as young as 7 years of age (Li et al. 2017). The highest frequencies of HFI are reported for postmenopausal women (Hershkovitz et al. 1999; Nikolić et al. 2010; Barber et al. 1997; Western and Bekvalac 2017; Djonic et al. 2016; Gershon‐Cohen et al. 1955; Salmi et al. 1962; Raikos et al. 2011; Verdy et al. 1978; Wilczak and Mulhern 2012), and in patients with acromegaly (Fulton et al. 1990; Littlejohn et al. 1986), an endocrine disorder that involves the overproduction of growth hormone. In males, HFI has been associated with Klinefelter's syndrome (Ramchandren and Liebeskind 2007), castration (Belcastro et al. 2011), testicular atrophy (Beatty et al. 2021), hypogonadism (Yamakawa et al. 2006), and chemical androgen suppression (May, Peled, Dar, Abbas, et al. 2010). The clinical significance of HFI is debated. Extensive lesions reduce intracranial volume (May et al. 2012), which may compress the brain and ultimately lead to irritation or soft tissue atrophy. Although there is extensive conjecture, the investigation of HFI as a correlate of neurological impairment (Byrne et al. 2020; Waclawik 2006; Talarico et al. 2008; Li et al. 2017; Djonic et al. 2016; Gilbert et al. 2009; De Zubicaray et al. 1997; Suzuki et al. 2017; Brodoehl et al. 2013; Korja et al. 2007), psychiatric illness (Hasegawa et al. 1983; Moore 1935; Chaljub et al. 1999; Devriendt et al. 2005; Djonic et al. 2016; Hawkins and Martin 1965; Varotto et al. 2023; Stewart 1928; Eldridge 1940; Elghazouani 2021), or other bone diseases (Djonic et al. 2016; Wilczak and Mulhern 2012; Littlejohn et al. 1986) has mainly been limited to case reports. A qualitative systematic review of case reports recently identified headaches, obesity, vertigo, cognitive decline, and depression as high‐frequency comorbidities of HFI (Alvarez et al. 2024).

**TABLE 1:** Summary of major findings across HFI studies.

Study	Study type	Method of observation	Location	Sex	Date	Total (*n*)	HFI (%)	Type A	Type B	Type C	Type D	Age range (years)^a^	Notes/Key findings
Alenezi et al. (2024)	Cross‐sectional study: Modern (Clinical)	PET/CT Scans—Hershkovitz et al. (1999)	Kuwait City, Kuwait	M	2019–2021 ce	23	9 (39%)	NR	NR	NR	NR	45–70	Only obese patients (BMI > 30) were included
Alenezi et al. (2024)	Cross‐sectional study: Modern (Clinical)	PET/CT Scans—Hershkovitz et al. (1999)	Kuwait City, Kuwait	F	2019–2021 ce	82	58 (70.7%)	NR	NR	NR	NR	45–70	Same as above
Barber et al. (1997)	Cross‐sectional study: Historic (Skeletal assemblages)	X‐ray Radiographs—Littlejohn et al. (1986)	North Lincolnshire, UK (Anglo‐Saxon)	M and F	12th–18th centuries ce	85	33 (38.8%)	n/a	n/a	n/a	n/a	35–40 (estimated avg.)	
Beatty et al. (2021)	Cross‐sectional study: Modern (Dissection)	Direct Observation—Hershkovitz et al. (1999)	New York, USA	M	2021 ce	14	7 (50.0%)	1 (7.1%)	1 (7.1%)	3 (21.4%)	2 (14.3%)	Adult	Males with HFI category C/D had testicular atrophy. Decreased numbers of interstitial cells (Leydig cells) were present in 83.3% of males with HFI.
Beatty et al. (2021)	Cross‐sectional study: Modern (Dissection)	Direct Observation—Hershkovitz et al. (1999)	New York, USA	F	2021 ce	22	21 (95.5%)	4	10	4	2	Adult	Missing data in the published paper.
Bracanovic et al. (2016)	Case–control study: Modern (Autopsies)	Direct Observation—Hershkovitz et al. (1999)	Belgrade, Serbia	F	2010s ce	20	n/a	4	4	4	8	58–81	Compared to the control group, women with HFI showed increased bone volume fraction, thicker and more plate‐like trabeculae, reduced trabecular separation and connectivity density in the diploe. Moreover, the inner table in women with HFI showed increased total porosity and mean pore diameter compared to controls.
Cvetković et al. (2019)	Case–control study: Modern (Autopsies)	Direct Observation—Hershkovitz et al. (1999)	Belgrade, Serbia	M	2007–2016 ce	35	n/a	7 (20%)	16 (45%)	9 (26%)	3 (9%)	27–85	Compared to the control group, frontal and temporal bones were thicker in all subjects who had HFI. Age seemed to be a predictive factor for HFI only in females. Females younger than 55 years have a similar risk for HFI occurrence as males.
Cvetković et al. (2019)	Case–control study: Modern (Autopsies)	Direct Observation—Hershkovitz et al. (1999)	Belgrade, Serbia	F	2007–2016 ce	112	n/a	12 (11%)	25 (22%)	46 (41%)	29 (26%)	24–93	Same as above
Cvetković et al. (2020)	Sex‐stratified analysis: Modern (Autopsies)	Direct Observation—Hershkovitz et al. (1999)	Belgrade, Serbia	M	2007–2016 ce	19	n/a	5 (26.3%)	6 (31.6%)	5 (26.3%)	3 (15.8%)	63–85	There is no difference in microarchitectural structure of the frontal bone between males and females with HFI, in general aspect and within corresponding HFI subtypes.
Cvetković et al. (2020)	Sex‐stratified analysis: Modern (Autopsies)	Direct Observation—Hershkovitz et al. (1999)	Belgrade, Serbia	F	2007–2016 ce	17	n/a	4 (23.5%)	4 (23.5%)	5 (29.4%)	4 (23.5%)	28–88	Same as above
Devriendt et al. (2005)	Cross‐sectional study: Modern (Autopsies)	Direct Observation—Hershkovitz et al. (1999)	France	M and F	1999–2002 ce	1532	13 (0.8%)	0 (0.0%)	0 (0.0%)	5 (38.5%)	8 (61.5%)	42–79	1 M, 12 F; documentation of psychiatric illness
Djonic et al. (2016)	Case–control study: Modern (Clinical)	CT Scans—Hershkovitz et al. (1999)	Belgrade, Serbia	F	2011–2014 ce	103	48 (46.6%)	28 (27.2%)		20 (19.4%)		60–76	
Du Fayet de la Tour et al. (2023)	Cross‐sectional study: Ancient (Cremains)	Direct Observation—Hershkovitz et al. (1999)	Pompei, Metropolitan City of Naples, Campania, Italy (Roman period)	M and F	1st century bce—1st century ce	71	11 (15.5%)	NR	NR	NR	NR	Young adult to older adult	90.9% of the individuals displayed degenerative conditions in conjunction with HFI
Eldridge (1940)	Cross‐sectional study: Modern (Clinical)	X‐ray Radiographs	District of Columbia, USA	F	1940s ce	200	50 (25%)	n/a	n/a	n/a	n/a	20–89	The sample only included female patients at a mental hospital
Flohr and Witzel (2011)	Cross‐sectional study: Prehistoric (Skeletal assemblages)	Direct Observation—Hershkovitz et al. (1999)	Qatna, Syria (Bronze age)	M and F	2700 bce	70 (MNI)	9 (12.8%)	2 (22%)	3 (33%)	1 (11%)	3 (33%)	Adult	Highly fragmentary remains
Flohr et al. (2023)	Cross‐sectional study: Historic (Skeletal assemblages)	Direct Observation—Hershkovitz et al. (1999)	Lower Saxony, Germany (Medieval period)	M and F	8th–11th centuries ce	83 (MNI)	1 (1.2%)	0 (0.0%)	0 (0.0%)	0 (0.0%)	1 (1.2%)	50+	Co‐occurrence with resorptive lesion of the sella turcica with dehiscence of the floor of the hypophyseal fossa
Fulton et al. (1990)	Case–control study: Modern (Clinical: hospital patients with acromegaly)	X‐ray Radiographs	Glasgow, UK	M and F	1980s ce	36	26 (72%)	n/a	n/a	n/a	n/a	30–83	HFI is strongly associated with acromegaly, especially in the presence of co‐existent hyperprolactinemia.
Fulton et al. (1990)	Case–control study: Modern (Clinical control group)	X‐ray Radiographs	Glasgow, UK	M and F	1980s ce	36	3 (25%)	n/a	n/a	n/a	n/a	Age matched to the above	Same as above
Gershon‐Cohen et al. (1955)	Case–control study: Modern (Clinical)	X‐ray Radiographs	Pennsylvania, USA	M	1950s ce	49	0 (0%)	n/a	n/a	n/a	n/a	58–96	
Gershon‐Cohen et al. (1955)	Case–control study: Modern (Clinical)	X‐ray Radiographs	Pennsylvania, USA	F	1950s ce	79	49 (62%)	n/a	n/a	n/a	n/a	58–96	HFI was not associated with virilism or obesity
Glab et al. (2006)	Cross‐sectional study: Historic (Skeletal assemblages)	Direct Observation—Hershkovitz et al. (1999)	Raciborz, Poland (Medieval period)	F?	16th century ce	11	2 (18.2%)	n/a	n/a	2	n/a	Older adults	Suspected kinship among individuals with HFI
Hajdu et al. (2009)	Case series: Mostly Historic (Skeletal assemblages, various)	Direct Observation—Hershkovitz et al. (1999)	Hungary	M and F	15th century bce—18th century ce	803 (412 M, 391 F)	20 (2.5%)	NR	NR	NR	NR	Older adults	
Hawkins and Martin (1965)	Cross‐sectional: Modern (Clinical)	X‐ray Radiographs	East Anglia, UK	M	1959–1963 ce	695	2 (0.3%)	n/a	n/a	n/a	n/a	< 25–85+	*n* (total) = 2031; *n* (HFI) = 103 (5.1%)
Hawkins and Martin (1965)	Cross‐sectional: Modern (Clinical)	X‐ray Radiographs	East Anglia, UK	F	1959–1963 ce	1336	101 (7.6%)	n/a	n/a	n/a	n/a	< 25–85+	Same as above
Hershkovitz et al. (1999)	Cross‐sectional study: Historic (Skeletal collection)	Direct Observation—Hershkovitz et al. (1999)	Hamann‐Todd Osteological Collection, Terry Collection	M	20th century ce	1007	52 (5.2%)	35 (3.5%)	16 (1.6%)	1 (0.1%)	0 (0.0%)	20–80+	Combined European and African American data
Hershkovitz et al. (1999)	Cross‐sectional study: Historic (Skeletal collection)	Direct Observation—Hershkovitz et al. (1999)	Hamann‐Todd Osteological Collection, Terry Collection	F	20th century ce	699	167 (23.9%)	17 (2.4%)	73 (10.4%)	46 (6.6%)	31 (4.4%)	20–80+	Same as above
Hershkovitz et al. (1999)	Cross‐sectional study: Historic (Skeletal collections)	Direct Observation—Hershkovitz et al. (1999)	National Museum of Natural History (NMNH) Collections, Smithsonian Institution	M and F	4th millennium bce–7th century ce	1012	0 (0.0%)	n/a	n/a	n/a	n/a	Adults	
Hershkovitz et al. (1999)	Cross‐sectional study: Modern (Dissection)	Direct Observation—Hershkovitz et al. (1999)	Jerusalem, Tel Aviv, Israel	M	1990s ce	35	7 (20.0%)	4 (11.4%)	1 (2.8%)	1 (2.8%)	1 (2.8%)	85.6 (avg.)	*n* (total) = 72; *n* (HFI) = 26 (36.1%)
Hershkovitz et al. (1999)	Cross‐sectional study: Modern (Dissection)	Direct Observation—Hershkovitz et al. (1999)	Jerusalem, Tel Aviv, Israel	F	1990s ce	37	19 (51.4%)	7 (18.9%)	3 (8.1%)	6 (8.1%)	3 (8.1%)	84.0 (avg.)	*n* (total) = 72; *n* (HFI) = 26 (36.1%)
Kollin and Feher (1986)	Case–control study: Modern (Clinical)	X‐ray Radiographs	Budapest, Hungary	F	1980s ce	81	n/a	n/a	n/a	n/a	n/a	20–45	All patients were pre‐menopausal. Greater than normal bone mineral content, bone width of the radius, and bone mineral content to bone width ratio was found in women with HFI. An increase in serum DHEAS, DHEA, and testosterone was also detected in HFI patients.
Littlejohn et al. (1986)	Case–control study: Modern (Clinical)	X‐ray Radiographs	Melbourne, Australia	M and F	1980s ce	30 (14 M, 16 F)	26 (87%)	n/a	n/a	n/a	n/a	18–87	All patients in the focal group had acromegaly. Sex specific data not reported.
May et al. (2012)	Cross‐sectional study: Modern (Clinical)	CT Scans	Haifa, Israel	F	2010s ce	380	278 (73.2)	n/a	n/a	n/a	n/a	60+	All patients were post‐menopausal. HFI was classified into minor, moderate, and severe types
May et al. (2010)	Case–control study: Modern (Prostate cancer patients treated with androgen blockers)	CT Scans	Haifa, Israel	M	2004–2007 ce	67	39 (58.2%)	n/a	n/a	n/a	n/a	78.54 (avg.)	Study group had prostate cancer.
May et al. (2010)	Case–control study: Modern (Prostate cancer patients NOT treated with androgen blockers)	CT Scans	Haifa, Israel	M	2004–2007 ce	60	25 (41.7%)	n/a	n/a	n/a	n/a	78.54 (avg.)	Males who received a complete androgen block had a significantly higher prevalence of HFI compared to healthy males. There is a positive association between the length of hormonal treatment and the manifestation of HFI.
May et al. (2010)	Case–control study: Modern (Clinical control group)	CT Scans	Haifa, Israel	M	2004–2007 ce	180	63 (35%)	n/a	n/a	n/a	n/a	77.82 (avg.)	Same as above
Moore (1935)	Cross‐sectional study: Modern (Clinical)	X‐ray Radiographs	Washington, USA	M and F	1910–1930s ce	5955	72 (1.2%)	n/a	n/a	n/a	n/a	44 (avg.)	*n* = 70 (97.2%) of cases were in females; *n* = 70 (97.2%) had patient histories of neurological or neuropsychiatric symptoms
Mulhern et al. (2006)	Cross‐sectional study: Historic (Skeletal collection)	Direct Observation—Hershkovitz et al. (1999)	New Mexico, USA (Chaco Canyon burials at NMNH)	M and F	860–935 ce	37	12 (32.4%)	2 (5.4%)	7 (18.9%)	3 (8.1%)	0 (0.0%)	20–60 (estimated)	1 out of 12 males (8.3%) and 11 out of 25 females (44.0%) had HFI
Nikolic et al. (2010)	Cross‐sectional study: Modern (Autopsies)	Direct Observation—Hershkovitz et al. (1999)	Belgrade, Serbia	M	2000s ce	1220	9 (0.74%)	3 (0.25%)	3 (0.25%)	3 (0.25%)	0 (0%)	28–80	Measured bone thickness; HFI increased, but severity was not correlated with age
Nikolic et al. (2010)	Cross‐sectional study: Modern (Autopsies)	Direct Observation—Hershkovitz et al. (1999)	Belgrade, Serbia	F	2000s ce	248	45 (18.1%)	7 (15.6%)	14 (31.1%)	19 (42.2%)	5 (11.1%)	19–93	Same as above
Ntlholang et al. (2014)	Cross‐sectional study: Modern (Clinical)	CT Scans—Modified Hershkovitz et al. (1999)	Dublin, Ireland	M	2014 ce	75	2 (2.7%)	n/a		n/a		69.9 (avg.)	
Ntlholang et al. (2014)	Cross‐sectional study: Modern (Clinical)	CT Scans—Modified Hershkovitz et al. (1999)	Dublin, Ireland	F	2014 ce	69	29 (42.0%)	19 (65.5%)		10 (35.5%)		70.1 (avg.)	
Raikos et al. (2011)	Cross‐sectional study: Modern (Skeletal collection)	Direct Observation—Hershkovitz et al. (1999)	Bochum, Germany	M and F	20th century ce	204	25 (12.3%)	7 (3.4%)	7 (3.4%)	6 (2.9%)	5 (2.5%)	20–94	
Raikos et al. (2011)	Cross‐sectional study: Modern (Dissection)	Direct Observation—Hershkovitz et al. (1999)	Bochum, Germany	M and F	2010s ce	40	5 (12.5%)	1 (2.5%)	0 (0%)	2 (5.0%)	3 (7.5%)	18–65+	22.7% in females, 0% in males. 87.5% of severe HFI cases were found in females over 65 years old
Salmi et al. (1962)	Cross‐sectional study: Modern (Clinical)	X‐ray Radiographs	Helsinki, Finland	M	1960s ce	334	9 (2.7%)	n/a	n/a	n/a	n/a	22–82	*n* (total) = 982; *n* (HFI) = 117 (11.9%)
Salmi et al. (1962)	Cross‐sectional study: Modern (Clinical)	X‐ray Radiographs	Helsinki, Finland	F	1960s ce	648	108 (16.7%)	n/a	n/a	n/a	n/a	22–82	HFI is more frequent in postmenopausal women (22%) vs. women of reproductive age (17%)
Szeniczey et al. (2019)	Cross‐sectional study: Ancient and historic (Skeletal assemblages)	Direct Observation—Hershkovitz et al. (1999)	Hungary and Serbia	M	4900 bce—17th century ce	2258	46 (2.0%)	42 (1.9%)	4 (0.2%)	0 (0%)	0 (0%)	20–50+ (estimated)	Study hypothesizes that the physiological effects of the pastoralist lifestyle and diet on insulin regulation could explain the increased risk of developing HFI in the 5th–8th and 10th‐century populations
Szeniczey et al. (2019)	Cross‐sectional study: Ancient and historic (Skeletal assemblages)	Direct Observation—Hershkovitz et al. (1999)	Hungary and Serbia	F	4900 BCE—17th century ce	2074	115 (5.5%)	106 (5.1%)	4 (0.2%)	2 (0.1%)	3 (0.1%)	20–50+ (estimated)	Same as above
Verdy et al. (1978)	Cross‐sectional study: Modern (Clinical)	X‐ray Radiographs	Montreal, Canada	F	1970s ce	263	127 (49.0%)	n/a	n/a	n/a	n/a	60–80	Study sample included nulliparous nuns. A prevalence of 84% of HFI was found in the obese subjects compared to 16% in the underweight subjects
Vukovic et al. (2020)	Cross‐sectional study: Modern (Clinical)	MRI Scans	Novi Sad, Serbia	M	2016–2019 ce	338	17 (5.0%)	n/a	n/a	n/a	n/a	18–81	HFI was associated with increased age in women, but not in men
Vukovic et al. (2020)	Cross‐sectional study: Modern (Clinical)	MRI Scans	Novi Sad, Serbia	F	2016–2019 ce	570	57 (10.0%)	n/a	n/a	n/a	n/a	18–91	Same as above
Western and Bekvalac (2016)	Cross‐sectional study: Historic (Skeletal assemblages)	Direct Observation/X‐ray Radiographs	London, UK (Industrial period)	M/F	1676–1852 ce	138	22 (15.9%)	n/a	n/a	n/a	n/a	20–60+	HFI increased with age at death and was associated with nulliparity; Ambiguous A/B lesion types can be confirmed with micro‐CT
Wilczak and Mulhern (2012)	Cross‐sectional study: Historic (Skeletal collection)	Direct Observation—Hershkovitz et al. (1999)	Terry Collection, NMNH, Smithsonian Institution	M	1899–1941 ce	212	21 (9.9%)	NR	NR	NR	NR	40–102	HFI was a significant determinant of DISH, but age was not
Wilczak and Mulhern (2012)	Cross‐sectional study: Historic (Skeletal collection)	Direct Observation—Hershkovitz et al. (1999)	Terry Collection, NMNH, Smithsonian Institution	F	1899–1941 ce	194	110 (56.7%)	NR	NR	NR	NR	40–102	Same as above

*Note:* Significance of a indicates known age ranges were given whenever possible. Parentheses indicate an estimated age range, as is sometimes the case for bioarchaeological samples, or the mean age if a range was not reported in the original paper.

HFI was previously thought to be a disease of modernity due to its rarity in bioarcheological assemblages. However, numerous studies have documented its occurrence before the 19th century (Rühli et al. 2004; Barber et al. 1997; Western and Bekvalac 2017; Belcastro et al. 2011; Bebel and Golijewskaja 2015; Shahin et al. 2014; Armelagos and Chrisman 1988; Hajdu et al. 2009; Laffranchi et al. 2020; Flohr and Witzel 2011; Flohr et al. 2023; Glab et al. 2006; Szeniczey et al. 2019; Mulhern et al. 2006; de la Du Fayet Tour et al. 2023), with an antiquity dating as far back as 1.5 million years (Antón 1997). Lower prevalence rates in past populations may be attributed to lower life expectancy (Rühli et al. 2004; Armelagos and Chrisman 1988), or to the less robust diagnostic methods that are sometimes used in paleopathology research (Barber et al. 1997). It remains plausible that HFI has become more common over time (Hershkovitz et al. 1999; Western and Bekvalac 2017; May, Peled, Dar, Abbas, and Hershkovitz 2011). Alternatively, one study posits that HFI prevalence may have fluctuated throughout different periods in response to changes in subsistence strategy (Szeniczey et al. 2019). Given high prevalence rates in postmenopausal females, HFI lesions have been used to infer sex and age in bioarcheological investigations involving burnt or fragmentary human skeletal remains (de la Du Fayet Tour et al. 2023). HFI may have similar utility in forensic casework when remains are badly damaged by post‐depositional processes (Cvetković et al. 2019; Devriendt et al. 2005; May, Peled, Dar, Cohen, et al. 2011).

The present research uses reliable demographic data and includes variables that are often difficult to assess in other anthropological studies (e.g., cause of death and ZIP code as a social indicator of health). By targeting an anatomical body donor population that includes a high percentage of older individuals, this work investigates whether geriatric females and males exhibit similar HFI prevalence and severity rates, and whether other demographic variables and comorbidities extracted from death certificates can be used as predictors of HFI.

## 2 Material and Methods

### 2.1 Study Sample

The sample includes 235 anatomical body donors used in teaching dissections from August 2023 to August 2025 at multiple medical schools throughout the Chicagoland area (e.g., Rush University Medical Center, University of Illinois at Chicago (UIC), Northwestern University). All donors were willfully consented to and supplied to member institutions by the Anatomical Gift Association of Illinois (AGAI). The AGAI is formally charged with acquiring, preparing, and distributing the bodies donated for anatomical study on behalf of most medical schools in the state of Illinois. While a summary of the body donor profile is not yet available for the Chicagoland area, several studies have shown that educated, White individuals over the age of 65 years are the most likely to bequeath their bodies for education and research purposes (Collins et al. 2018; Mueller et al. 2021; Bagian et al. 2024). Only donors who underwent craniotomies could be included in the study. For the most part, craniotomies were performed at the AGAI and based on the needs of each dissection program. Thus, they were completely independent of donor history.

The donor demographic data extracted for this study include information on known sex, age at death, date of death, cause of death (COD), marital status, and ZIP code. This information was not available upon initial assessment of HFI and was subsequently retrieved from death records curated at the AGAI after HFI data collection was complete. Unfortunately, unlike many other states, the Illinois death certificate does not consistently document socially ascribed race, occupation, or educational status.

COD was coded using the World Health Organization's (WHO) International Classification of Diseases (ICD‐11) for Mortality and Morbidity Statistics (World Health Organization 2022). Likewise, each ZIP code was assigned a social vulnerability index (SVI) (Kim and Bostwick 2020). The SVI is a measurement tool that was initially developed by the Centers for Disease Control (CDC) to assess the susceptibility of a community or population to the negative impacts of external factors like disease outbreaks or natural disasters, based on pre‐existing systemic inequalities and social structures. It remains a critical tool in understanding the social determinants of health. Given that lived social inequities also influence dental and skeletal biomarkers, researchers are beginning to advocate for the application of a structural vulnerability framework in medicolegal reporting (Znachko et al. 2023; Winburn et al. 2022). Using data from the U.S. Census American Community Survey, the UIC School of Public Health developed an SVI for the state of Illinois to determine risk during the COVID‐19 pandemic (Kim and Bostwick 2020). This index considers the percent of residents living in poverty, the percent of residents with less than a high school education, the percent of households headed by a female, the median household income, and employment ratios. These indicators are strongly correlated with an individual's health status and vary markedly across the Chicagoland area (Kim and Bostwick 2020). To contextualize SVI findings in the donor population, an Illinois‐wide reference distribution was created. SVI values across all Illinois ZIP codes, weighted by population size, were aggregated using publicly available data from the U.S. Census Bureau (U.S. Census Bureau 2020).

### 2.2 Macroscopic Methods

**FIGURE 1:**
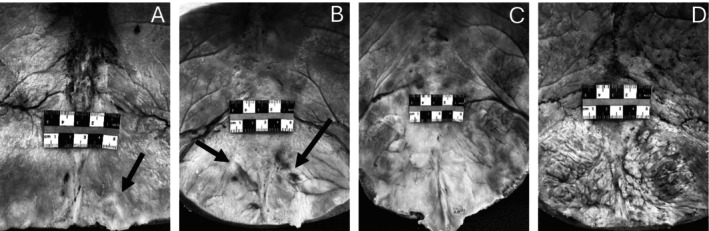
HFI varies in shape and size. HFI type A (A) demonstrates isolated bony islands that are < 10 mm. They can be either single or multiple. Only slight elevation in noted in this individual (arrow). HFI type B (B) is characterized by multiple bony nodules, often without discrete margins (arrows), on less than 25% of the endocranial surface of the frontal bone. HFI type C (C) shows more extensive lesions comprising up to 50% of the frontal bone and is associated with an irregular thickening of the inner bone table. HFI type D (D) involves the continuous bony overgrowth of over 50% of the endocranial surface of the frontal bone. Lesions are elevated with irregular, but clearly demarcated borders.

The prevalence and severity of HFI were assessed using the classification system developed by Hershkovitz et al. (1999). This system defines four stages of HFI development based on its morphological appearance and the size of the affected area. Type A includes isolated lesions that are less than 10 mm in size and progresses to types B, C, and D as the nodules become larger and incorporate more of the frontal bone (i.e., < 25%, 25%–50%, and > 50% respectively) (see Figure 1). These percentages only pertain to the frontal squama as the orbital portion of the frontal bone does not appear to be affected (Moore 1935). When possible, notes on any gross features that have previously been associated with HFI (e.g., pituitary hypertrophy, hyperostosis cranialis diffusa, testicular atrophy) were recorded (Fulton et al. 1990; Littlejohn et al. 1986; Belcastro et al. 2011; Yamakawa et al. 2006).

### 2.3 Statistical Methods

#### 2.3.1 Donor Demographics

Descriptive statistics were calculated for all variables. Normality was assessed using Shapiro–Wilk tests. All continuous variables were non‐normally distributed, resulting in the use of non‐parametric statistics. Chi‐square tests were used to test for significant differences in marital status, birthplace, and COD between females and males. Mann–Whitney U tests examined sex differences in age‐at‐death and SVI. A cumulative distribution function (CDF) plot was created to illustrate the differences in SVI between the anatomical body donor population and the greater population of the state of Illinois. A Monte Carlo simulation (100,000 iterations), using weighted random sampling, was then conducted to estimate the distribution of mean SVIs for the Illinois population using a sample size equal to that of the donor population (*n* = 235). To assess whether the donor SVI was significantly different from the state population, a two‐tailed Z‐test was performed. The relationship between COD with sex, age‐at‐death, and SVI was then explored using binomial logistic regression analysis. The models were built with one dependent variable (COD) and multiple independent variables (sex, age‐at‐death, and SVI as indicators).

#### 2.3.2 HFI Prevalence and Severity

The prevalence and severity of HFI between females and males were compared using chi‐square tests. Relationships between HFI and continuous demographic variables were explored through Spearman's rank correlation. Binomial logistic regression was used to test for differences in HFI presence by COD category. The model was built with one dependent variable (HFI prevalence) and multiple independent variables (each COD as the main variable of interest and sex and age‐at‐death as confounding variables). In instances where donors had multiple CODs reported, all CODs were used in the analysis. All statistical analyses were performed in SPSS or in Python (scipy.stats, statsmodels.api).

#### 2.3.3 Research Approval

This project received written approval from the Board of Directors of the AGAI and was deemed IRB exempt by Rush University Medical Center.

## 3 Results

### 3.1 Donor Demographics

**FIGURE 2:**
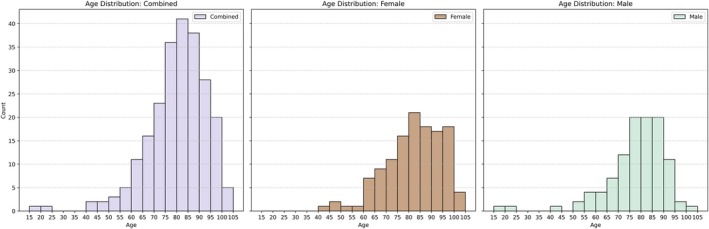
Age‐at‐death distribution by sex.

**FIGURE 3:**
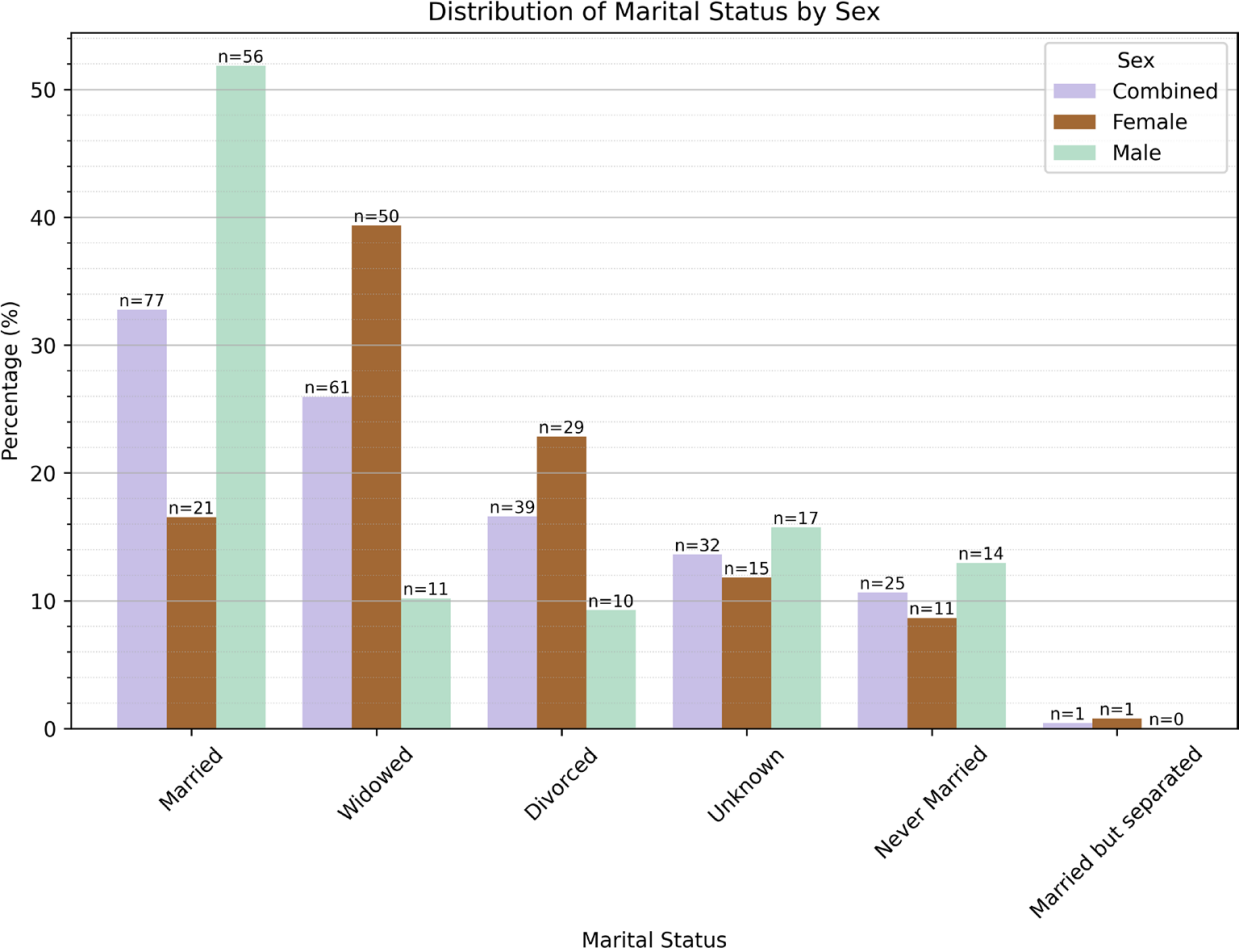
Marital status distribution by sex.

**FIGURE 4:**
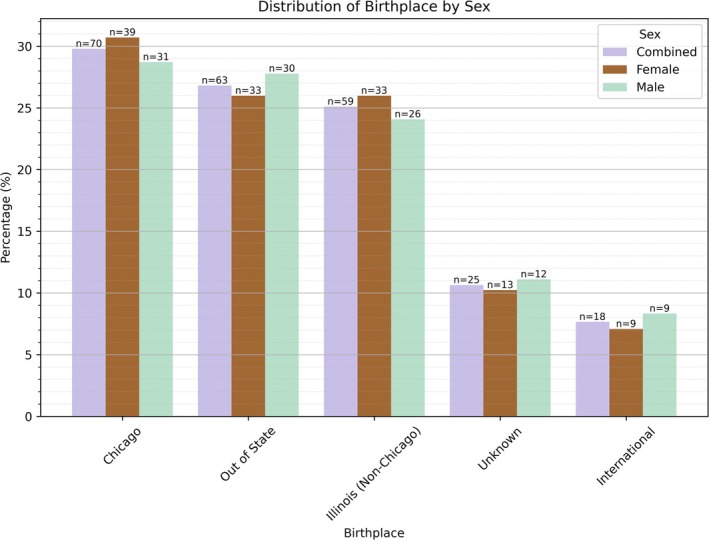
Birthplace distribution by sex.

**FIGURE 5:**
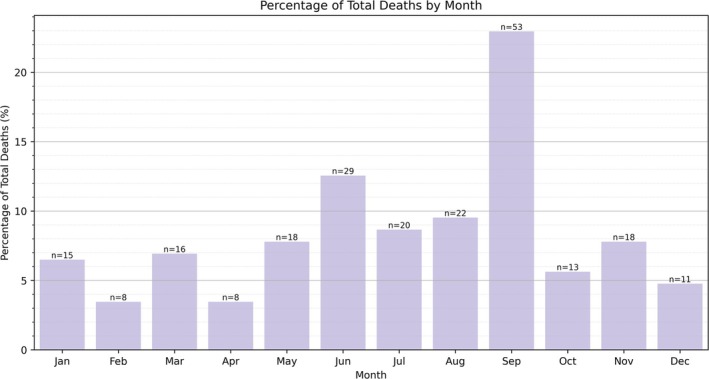
Month of death distribution for the total sample.

Females were slightly overrepresented in the study sample, accounting for 127 individuals (54.0%), while males comprised 108 individuals (46.0%). Figure 2 displays the age‐at‐death distribution for the sample, with a mean of 79.9 years. A Mann–Whitney *U* test revealed a statistically significant difference in age‐at‐death between sexes (*U* = 5497.5, *Z* = −2.319, *p* = 0.0203), indicating a survivorship advantage for females, who lived about 4 years longer on average. Marital status for the sample is shown in Figure 3. A chi‐square test revealed significant sex differences (*χ* ^2^ [5, *N* = 235] = 50.4, *p* = 1.16e‐9). Standardized residuals suggest that males are more likely to die with a surviving spouse (standardized residual = 3.47, *p* = 5.20e‐4), whereas females are more likely to die as widows (standardized residual = 2.97, *p* = 2.98e‐3). Figure 4 shows the distribution of birthplace in the sample, revealing no significant differences between females and males (*χ* ^2^ [4, *N* = 235] = 0.394, *p* = 0.983). A large portion of the study sample was born in Chicago (29.8%), followed by states other than Illinois (26.8%), other parts of Illinois (25.1%), and internationally (7.7%). Birthplace information was unknown for the remaining 10.6%. The month of death for the total sample is shown in Figure 5. The disproportionate number of September deaths most likely reflects a sampling bias rather than a true phenomenon. The University of Chicago receives its body donors with intact cranial cavities, and coincidentally, its anatomy program has an earlier dissection schedule than other AGAI member institutions. Thus, these individuals were not included in our analyses.

**FIGURE 6:**
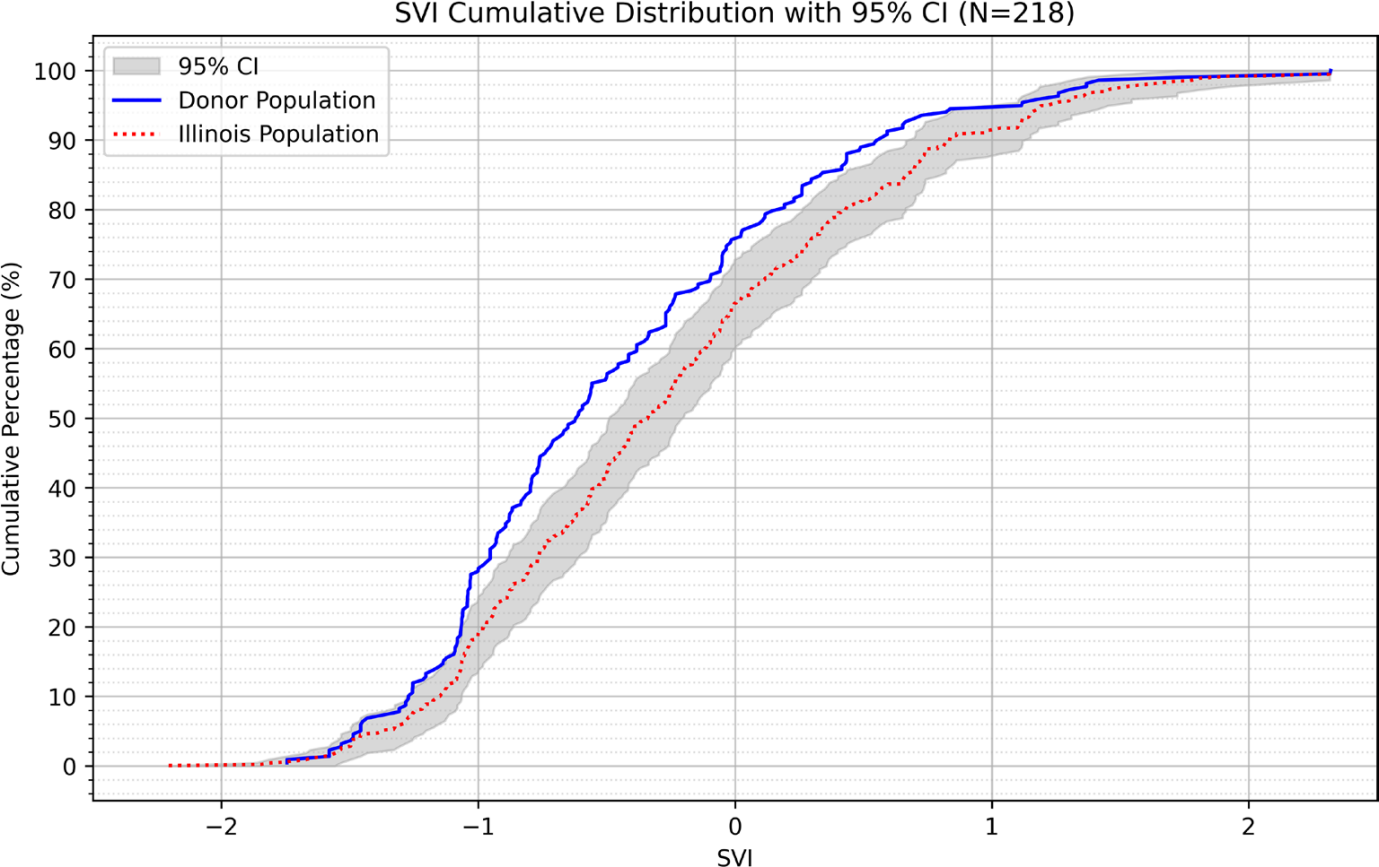
SVI Cumulative distribution of the donor and great Illinois populations with Monte Carlo simulation.

Seventeen donors either died outside of the state of Illinois (mostly Indiana) or had unknown Zip codes and were therefore excluded from SVI analyses. The cumulative distribution function (CDF) plot of SVIs is shown in Figure 6. The anatomical body donor population had a mean SVI of −0.452. The estimated mean SVI of the population of Illinois was −0.240, with a standard deviation of 0.0546 across the Monte Carlo simulation. The difference between the donor and state populations was significant (*Z* = −3.88, *p* = 1.05e‐4). Within the donor population, SVI also significantly differed between females and males (*U* = 4836.5, *Z* = −2.214, *p* = 0.027). Compared to females (*μ* = −0.587), males (*μ* = −0.308) demonstrated higher structural vulnerability risk. The frequency of the immediate COD is shown in Table 2. Neoplasms (23.0%) and diseases of the circulatory system (19.6%) were the most common CODs, followed by symptoms, signs, or clinical findings, not elsewhere classified (14.5%), and diseases of the respiratory system (11.1%). Other CODs were relatively rare (≤ 6%). Summary statistics from all comparisons between COD categories, sex, age‐at‐death, and SVI using binomial logistic regression are detailed in Table 3. Diseases of the respiratory system (COD12) was the only COD category where SVI emerged as a statistically significant predictor of mortality. A higher SVI was associated with an increased likelihood of dying from a respiratory illness as either an immediate or underlying COD. There were no significant differences in CODs between females and males. Neoplasms (COD02) and diseases of the digestive system (COD13) were associated with a lower age‐at‐death, whereas diseases of the nervous (COD08) and circulatory systems (COD11) were associated with a higher age‐at‐death. The odds ratio for these effects ranged from 0.957 to 1.067 per year.

**TABLE 2:** Descriptive statistics for the study sample (*n* = 235) by immediate cause of death.

Cause of death category	Number (%)
01 Certain infectious or parasitic diseases (e.g, sepsis)	8 (3.4%)
02 Neoplasms (e.g., cancer, adenocarcinoma, glioblastoma, leukemia, lymphoma, sarcoma)	54 (3.0%)
05 Endocrine, nutritional or metabolic diseases (e.g., protein calorie malnutrition)	3 (1.3%)
06 Mental, behavioral, or neurodevelopmental disorders (e.g., dementia [Alzheimer's, vascular, frontotemporal, Lewy Body], bipolar disorder)	14 (6.0%)
08 Diseases of the nervous system (e.g., cerebrovascular disease, stroke, Alzheimer's, Parkinson's, ALS)	32 (13.6%)
11 Diseases of the circulatory system (e.g., congestive heart failure, coronary artery disease, myocardial infarction, pulmonary embolism)	46 (19.6%)
12 Diseases of the respiratory system (e.g., respiratory failure, COPD, pneumonia)	26 (11.1%)
13 Diseases of the digestive system (e.g., bowel ischemia, cholecystitis, peritonitis, cirrhosis, liver failure)	6 (2.6%)
16 Diseases of the genitourinary system (e.g., chronic kidney disease, renal failure)	3 (1.3%)
21 Symptoms, signs, or clinical findings, not elsewhere classified (e.g., Adult failure to thrive, multiple organ failure, senile degeneration of the brain, sudden cardiac arrest/death, shock)	39 (16.6%)
22 Injury, poisoning or certain other consequences of external causes (e.g., asphyxiation, injury from a fall, pathological fracture)	4 (1.7%)
Total	235 (100%)

**TABLE 3:** Summary statistics from all comparisons between COD categories, sex, age‐at‐death, and SVI using binomial logistic regression.

Response	Indicator	Coefficient (*B*)	*z*	*p*	Odds ratio
COD01	Intercept	−2.6667	—	—	—
	SVI	0.4046	1.06	0.289	1.498693
	Age‐at‐death	−0.0006	−0.025	0.980	0.999391
	Sex	−0.1651	−0.256	0.798	0.847837
COD02	Intercept	2.097	—	—	—
	SVI	0.1789	0.862	0.389	1.195892
	Age‐at‐death	−0.0352	−2.919	0.004^a^	0.965404
	Sex	−0.5068	−1.549	0.121	0.602438
COD05	Intercept	−5.2755	—	—	—
	SVI	−0.5675	−0.934	0.350	0.566914
	Age‐at‐death	0.0212	0.633	0.527	1.021425
	Sex	−0.1707	−0.224	0.823	0.843085
COD06	Intercept	−5.2769	—	—	—
	SVI	−0.3774	−0.908	0.364	0.68564
	Age‐at‐death	0.0316	1.248	0.212	1.032102
	Sex	−0.1713	−0.31	0.756	0.842583
COD08	Intercept	−5.6533	—	—	—
	SVI	−0.0887	−0.351	0.725	0.915081
	Age‐at‐death	0.0445	2.408	0.016^a^	1.045537
	Sex	0.7366	1.913	0.056	2.08877
COD11	Intercept	−3.6936	—	—	—
	SVI	0.1049	0.491	0.623	1.110591
	Age‐at‐death	0.0359	2.499	0.012^a^	1.036597
	Sex	−0.3411	−1.057	0.29	0.710991
COD12	Intercept	−2.4507	—	—	—
	SVI	0.5728	2.263	0.024^a^	1.773144
	Age‐at‐death	0.0118	0.704	0.482	1.011829
	Sex	−0.2867	−0.687	0.492	0.750707
COD13	Intercept	0.7728	—	—	—
	SVI	0.1244	0.284	0.776	1.132451
	Age‐at‐death	−0.0441	−2.175	0.030^a^	0.95687
	Sex	−0.9967	−1.351	0.177	0.369083
COD16	Intercept	−6.2008	—	—	—
	SVI	−0.2732	−0.551	0.582	0.760974
	Age‐at‐death	0.0235	0.693	0.489	1.023801
	Sex	1.4657	1.728	0.084	4.330691
COD21	Intercept	−0.9208	—	—	—
	SVI	−0.1729	−0.669	0.504	0.841236
	Age‐at‐death	−0.0135	−0.965	0.334	0.986585
	Sex	0.388	0.999	0.318	1.474049
COD22	Intercept	−1.2491	—	—	—
	SVI	0.0573	0.111	0.912	1.058922
	Age‐at‐death	−0.0351	−1.46	0.144	0.965509
	Sex	0.6988	0.779	0.436	2.011251

*Note:* Several *p* values are statistically significant. These include age‐at‐death for COD 2 COD 8, COD 11, and COD 13, as well as SVI for COD 12. Statistically significant values now appear to be demarcated by footnote “a”.

^a^ indicate statistically significant results.

### 3.2 HFI Prevalence and Severity

**FIGURE 7:**
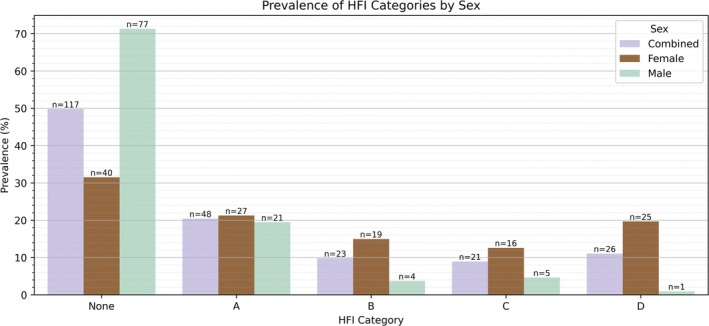
Prevalence of HFI category by sex.

The prevalence of HFI by sex and age‐at‐death is detailed in Table 4. Figure 7 illustrates the distribution of HFI in the study sample. The overall prevalence of HFI was 50.2%. Results suggest that there is a statistically significant difference in the distribution of HFI between females and males (*χ* ^2^ [4, *N* = 235] = 48.93, *p* = 6.030e‐10). HFI lesions of all types are more common in females than in males. Additionally, standardized residuals indicate that females are more likely to exhibit the most severe form of HFI compared to males (standardized residual = 2.92, *p* = 0.0035). However, there does not appear to be a strong relationship between HFI presence and age‐at‐death in females (*r*_*s*_ = −0.103, *p* = 0.255), males (*r*_*s*_ = 0.048, *p* = 0.625), or the total sample (*r*_*s*_ = 0.033, *p* = 0.616). HFI presence was also not associated with SVI in females (*r*_*s*_ = 0.013, *p* = 0.891), males (*r*_*s*_ = 3.72e‐4, *p* = 0.997), or the total sample (*r*_*s*_ = −0.043, *p* = 0.530).

**TABLE 4:** Descriptive HFI statistics for the study sample (*n* = 235) by sex.

HFI severity	Females		Males	
	Number (%)	Mean age in years (range)	Number (%)	Mean age in years (range)
None	40 (31.5%)	82.3 (44–101)	77 (71.3%)	77.5 (19–104)
Type A (mild)	27 (21.3%)	81.9 (47–98)	21 (19.4%)	79.7 (58–94)
Type B	19 (15.0%)	79.3 (49–97)	4 (3.7%)	78.8 (73–87)
Type C	16 (12.6%)	84.3 (71–96)	5 (4.6%)	73.2 (52–98)
Type D (severe)	25 (19.7%)	80.4 (62–102)	1 (0.9%)	88
Total	127 (100%)	81.7 (44–102)	108 (100%)	77.9 (19–104)

HFI logistic regression results are shown in Table 5. The binary regression model demonstrated an overall classification accuracy of 68.1%, with a sensitivity (true positive rate) of 67.5% and specificity (true negative rate) of 68.7%. The results indicated that sex was a strong and significant predictor of HFI presence (*B* = 1.52, SE = 0.305, *p* = 6.33e‐7). The odds ratio (OR = 4.572, 95% CI: 2.514–8.315) suggests that females were over four times more likely than males to have HFI. Similarly, neoplasms (COD02) emerged as a significant predictor (*B* = −1.061, SE = 0.446, *p* = 0.018), where individuals with this COD had a higher likelihood of HFI presence (OR = 0.346, 95% CI: 0.144–0.834). Given this potential link, cancer type and frequency are subsequently detailed in Table 6. Lung (*n* = 9) and breast (*n* = 7) cancer were the two most common neoplasms listed as either the immediate or underlying COD. Of the cancer‐related deaths, 14 individuals (22.6%) died from a hormone‐sensitive cancer, while 48 individuals (77.4%) died from a non‐hormone‐sensitive cancer. Breast, uterine, prostate, ovarian, testicular, osteosarcoma, and thyroid cancers were classified as hormone‐sensitive (Henderson and Feigelson 2000). A post hoc binomial regression analysis revealed a higher prevalence of HFI in hormone‐sensitive cancers compared to non‐hormone‐sensitive cancers, although this difference was not statistically significant (Table 7).

**TABLE 5:** Binomial logistic regression model results of HFI prevalence with COD, age, and sex as indicators.

Indicator	Coefficient (*B*)	*z*	*p*	Odds ratio
Intercept	−0.311	—	—	—
COD01	−0.838	−1.193	0.233	0.433
COD02	1.078	2.464	0.014^a^	2.938
COD05	0.283	0.364	0.715	1.327
COD06	0.453	0.746	0.456	1.574
COD08	0.081	0.170	0.865	1.085
COD11	0.473	1.145	0.252	1.605
COD12	0.010	0.020	0.983	1.010
COD13	1.179	1.467	0.143	3.250
COD16	−0.804	−0.868	0.385	0.448
COD21	−0.470	−1.076	0.282	0.625
COD22	−0.199	−0.210	0.834	0.820
Age‐at‐death	0.008	0.623	0.533	1.008
Sex	−1.510	−4.970	6.70e‐7^a^	0.221

*Note:* The *p* value for COD 02 and sex are statisically significant. Statistical significance now appears to be indicated by the footnote “a”.

^a^ indicate statistically significant results at the 0.05 level.

**TABLE 6:** Descriptive statistics for all neoplasms listed as a COD (*n* = 62).

Cancer type	Number (%)
Brain	4 (6.5%)
Bile Duct	2 (3.2%)
Breast^a^	7 (11.3%)
Bronchus/Lung	9 (14.5%)
Cervix^a^	1 (1.6%)
Bile Duct	2 (3.2%)
Colon	2 (3.2%)
Gallbladder	1 (1.6%)
Kidney	1 (1.6%)
Leukemia	3 (4.8%)
Lymphoma	4 (6.5%)
Myeloma	1 (1.6%)
Oropharynx	1 (1.6%)
Other	5 (8.1%)
Ovary^a^	2 (3.2%)
Pancreas	7 (11.3%)
Prostate^a^	4 (6.5%)
Rectum	1 (1.6%)
Sarcoma	2 (3.2%)
Skin	1 (1.6%)
Stomach	3 (4.8%)
Tongue	1 (1.6%)
Total	62 (100%)

^a^ Hormone‐sensitive neoplasms.

**TABLE 7:** Binomial logistic regression model results of HFI prevalence for COD02 (neoplasms) with hormone sensitivity, age, and sex as indicators.

Indicator	Coefficient (*B*)	*z*	*p*	Odds ratio
Intercept	−1.318	—	—	—
Hormone sensitivity	0.526	0.676	0.499	1.692
Age‐at‐death	0.036	1.230	0.2189	1.036
Sex	−1.857	−3.071	0.0021^a^	0.156

^a^ indicate statistically significant results at the 0.05 level.

## 4 Discussion

This work aimed to characterize HFI in an anatomical body donor population, assessing whether disease prevalence and severity varied according to demographic and comorbid risk factors, including sex, age‐at‐death, SVI, and COD. The study sample predominantly consisted of older individuals from the Chicagoland area who had a low SVI and died of natural causes. The two leading CODs in the state of Illinois are heart disease and cancer (Center for Disease Control 2022), which is mirrored in our results. Lung cancer is the leading cause of cancer‐related death in Illinois in both females and males, followed by breast and prostate cancer, respectively (Cancer Burden in Illinois 2025). Again, this is in broad agreement with our findings. The mean age‐at‐death was slightly higher than the state average of 77.1 years (Center for Disease Control 2022), likely reflecting both the minimum age requirement for anatomical gift giving and a relatively privileged donor population. Compared to the Illinois population, the study sample had a significantly lower SVI, indicating a donor population with better access to resources, stronger infrastructure, higher socioeconomic status, and greater overall resilience in coping with adverse events. This bias may be attributed to a general distrust of healthcare and biomedical research among some disadvantaged groups. Due to a long history of exploitation and discrimination, individuals from these communities may be less inclined to become anatomical body donors (Boulware et al. 2004; Scharf et al. 2010; Siminoff et al. 2006).

Mortality from diseases of the respiratory system (COD12) was associated with a higher SVI, implying greater exposure to socioeconomic hardships and an increased risk of disease. In the study sample, respiratory CODs were mainly limited to respiratory failure, chronic obstructive pulmonary disease (COPD), and pneumonia. While the SVI was generally low across the study sample, socioeconomic disparities in pulmonary health are well documented (Rocha et al. 2019; Gershon et al. 2012). Despite air quality improvements and decreased smoking rates, these disparities appear to have worsened over time (Gaffney et al. 2021).

There does not appear to be a relationship between HFI and either age‐at‐death or SVI in females, males, or the total sample. This is not altogether surprising given the limited range of variation within these demographic variables for the study sample. Both HFI prevalence and severity were greater in females than in males. The 68.5% prevalence rate of HFI in females is consistent with other cross‐sectional studies that examined older‐aged female populations within clinical contexts (Nikolić et al. 2010; Djonic et al. 2016; Gershon‐Cohen et al. 1955; Wilczak and Mulhern 2012; May et al. 2012; Alenezi et al. 2024) (see Table 1). Given the geriatric population examined, it is reasonable to conclude that older age is a risk factor for HFI in women, but prevalence does not necessarily continue to rise with age in postmenopausal groups. The 28.7% prevalence rate in older aged males is more difficult to interpret as it falls both above (Hershkovitz et al. 1999; Gershon‐Cohen et al. 1955; Ntlholang et al. 2014) and below (May, Peled, Dar, Abbas, et al. 2010) what has been reported elsewhere. HFI in males was largely confined to the earliest stages of disease progression, with only a single observed case of Type D. Type A and B lesions are often discrete and can be particularly challenging to identify on radiographs due to issues of superimposition (Western and Bekvalac 2017; Hershkovitz et al. 1999; May, Peled, Dar, Hay, et al. 2010). Moreover, these early lesions may be overlooked as non‐specific endostosis (Devriendt et al. 2005). Thus, HFI prevalence may be underreported in the literature, especially in men.

Despite a large body of literature, HFI remains largely unexplained. It has been reported in association with various metabolic, endocrinological, and neuropsychiatric conditions. The high prevalence of HFI in postmenopausal women in the present study reinforces the possibility of a hormone‐related cause. We further enhance the understanding of HFI by considering COD. Our findings indicate an increased prevalence of HFI among individuals who died from cancer. Although encouraging, these results should be interpreted with caution, given the study's relatively small sample size and the limited data resolution available from death certificates. A major limitation of this approach is that COD is not reflective of the full patient history, and pertinent comorbidities are not always captured. Additionally, the heterogeneity of cancer types, stages, and treatments among individuals in the sample may confound associations and limit the generalizability of these findings.

The correlation with cancer is nonetheless noteworthy because it could partly explain the higher prevalence rates of HFI in modern compared to past populations (Hershkovitz et al. 1999; Western and Bekvalac 2017; May, Peled, Dar, Abbas, and Hershkovitz 2011). Global cancer rates have dramatically increased over the last 50 years, largely because of demographic shifts in longevity (Weir et al. 2015; Lin et al. 2021; Wild et al. 2020). While numerous case reports have noted HFI in the presence of cancer (Prescher and Adler 1993; Laffranchi et al. 2020; Tripathi et al. 2012; Kang et al. 2016; Lai and Tomer 2021; Takahashi et al. 2024; Moreno‐Ballesteros et al. 2021; Behshad et al. 2015), a shared pathophysiological pathway remains tenuous. The linkage could be through indirect means, as many cancers can result in hormonal imbalances and/or may be treated with endocrine therapy (Mitra et al. 2022; Folkerd and Dowsett 2010; Fuentes et al. 2021; Satpathi et al. 2023). Interestingly, one study found no differences in HFI prevalence between men with prostate cancer and the control group, but significantly higher rates of HFI were observed in men receiving androgen deprivation therapy as part of their cancer treatment (May, Peled, Dar, Abbas, et al. 2010). Although not statistically significant, HFI appeared more prevalent among individuals with hormone‐sensitive cancers than those with non‐hormone‐sensitive cancers in our study. Additional research is needed to validate this finding and to further explore the underlying mechanisms. Considering the potential link between HFI and neoplasms, metastatic skull tumors should be included in the differential diagnosis (Tripathi et al. 2012; Kang et al. 2016; Lai and Tomer 2021; Takahashi et al. 2024; Moreno‐Ballesteros et al. 2021; Behshad et al. 2015). Metastatic tumors can present in various forms, appearing as purely lytic, purely sclerotic, or a combination of both lesion types. However, sclerotic skull vault metastases are very rare, and given their invasive nature, these malignancies rarely affect only the inner bone table (Pons Escoda et al. 2020; Kakkar et al. 2016). Benign tumors like button osteomas develop on the ectocranial bone and typically exhibit a well‐organized lamellar microstructure that is indistinguishable from normal bone tissue (Eshed et al. 2002). HFI may also be confused with other hypertrophic diseases, such as localized Paget's disease. Advanced Paget's disease involves the loss of separation between the cortical and trabecular bone. It is characterized by a distinctive patchwork pattern of woven and lamellar bone, intermixed with irregular cement lines (Seitz et al. 2009; De Boer et al. 2013). The study of bone microstructure may be useful for distinguishing HFI lesions from those manifest in other cranial pathologies (Ruhli et al. 2007; Fraberger et al. 2021).

Histological investigations are further warranted to better describe disease progression and to define its phases of development. One study concluded that the compact lamellar structure found in the inner bone table of an HFI specimen is indicative of a slow‐growing process (Ruhli et al. 2007). Some research efforts have begun to characterize bone microstructure in HFI using a μCT approach. In individuals with advanced HFI, these studies suggest increased bone density and cortical porosity in the inner bone table, as well as changes to trabecular bone organization in the diploë (Djonic et al. 2016; Flohr and Witzel 2011; Ruhli et al. 2007; Bracanovic et al. 2016; Cvetković et al. 2020). This represents a promising area of future HFI research as microstructural changes beyond the inner bone table appear likely.

Overall, our findings suggest that being female and having cancer as a COD significantly increases the likelihood of HFI presence. Given the high observed frequencies, increased awareness of this condition is warranted, as HFI can have clinical significance and may mimic other pathologies. This research also has important bioarcheological and forensic implications, as HFI has been used to estimate age and sex in unidentified skeletal remains due to its high prevalence in postmenopausal women. This assumption appears valid, especially in cases exhibiting severe HFI progression. Future studies may investigate more targeted patient histories to enhance predictive accuracy.

## Data Availability

The data that support the findings of this study are available on request from the corresponding author. The data are not publicly available due to privacy or ethical restrictions.
